# A Practical Treatise on Phthisis Pulmonalis

**Published:** 1861-06

**Authors:** 


A Practical Treatise on Phthisis Pulmonalis;—Embracing its Pathology, Causes, Symptoms, and Treatment. By L. M. Lawson, M. D., Profes or of Clinical Medicine in the University of Louisiana, etc., etc. Cincinnati: Hickey, Mallory & Co. New York: S. S. & W. Wood. 1861.

In a former number of the Examiner we simply announced the publication of the above work, with a promise to note its contents more in detail at some future time. That promise we now intend to fulfil. The work of Dr. Lawson, is a good sized octavo volume of 557 pages, printed on plain type, and substantially bound. It is divided into four parts, viz: Pathology of Phthisis; Etiology of Phthisis; Semeiology of Phthisis; and Therapeutics of Phthisis. The first part embraces thirteen chapters, in which the author discusses at considerable length, the nature of the "tuberculous constitution;" the "precursory stages of Phthisis;" pathological anatomy of the tubercle, its distribution, its successive changes, and the coincident condition of the blood and secretions; the secondary and tertiary lesions, and the varieties of Phthisis. The chief appreciable elements that make the

"tuberculous constitution," are, irregular or imperfect digestion; feebleness in the mechanical movements of respiration; a peculiarly delicate or attenuated condition of the capillaries; and an unduly sensitive condition of the nervous system, more especially that part of it connected with organic life.

In reference to enfeebled respiratory movements, the author says: "The special and evident change which occurs, consists in *impaired power of expansion.* At a more or less early period, the muscles concerned in the active expansion of the chest seem to lose a portion of their power, and the chest expands imperfectly and irregularly. In consequence of this, the *ordinary* breathing capacity is diminished, the respiratory murmur becomes more or less weakened, and, at the same time, wavy and even jerking. These changes in the respiratory murmur are readily perceived by ausculation, and the diminished capacity may not only be inferred from the condition of respiration, but positively demonstrated by the movements of the chest. I have long been in the habit of observing these modifications of the respiratory function, and regard them as decidedly the most characteristic of all the signs supposed to indicate the existence of this state of the constitution."

The imperfect expansion of the chest here described by Dr. Lawson, together with the altered or "wavy" respiratory murmur, we have also noticed and studied carefully for several years. But, like Dr. Thompson, we have regarded it as indicative of the first step in the progress of actual tuberculosis, rather than as evidence of the mere tuberculous constitution. And we have felt the more confident in this view, from the fact that, we have almost invariably found the diminished expansion and wavy murmur, connected with increased vibration of voice and an appreciable diminution of resonance on percussion; signs of increased density, which could hardly result from simple feebleness of expansion.

In reference to the imperfect development of the capillaries and other structures, the author says: "The pulmonary system, embracing the areolar tissue and capillaries, partakes of the same defects that belong to these structures in the tuberculous constitution generally; hence, it may be assumed that the capillaries of the lungs are comparatively weak and attenuated, and the areolar tissue coarse and inelastic."

The smaller size and diminished contractility of the pulmonary capillaries in the tuberculous constitution, have been pointed out by Mr. Ancell, and, at least, partially demonstrated by Dr. J. S. Campbell. If we grant this defective condition of the capillaries, coupled, as it is, with undue nervous excitability, or perhaps more properly, exalted organic susceptibility, we shall readily perceive why persons with this constitution are so sensitive to morbid impressions, and especially to atmospheric changes. We have arrested the attention of the reader, here, upon the very threshold of Dr. Lawson's work, simply because a clear comprehension of the nature of the tuberculous constitution, and the stage of predisposition, is of the utmost practical value. It is the time when we may adopt judicious hygienic regulations and remedial measures with a prospect of success.

Part II, embraces three chapters, namely: one on *hereditary* predisposition; one on "causes which may induce Phthisis, independent of an hereditary predisposition;" and one on the "pathological inducing causes of Phthisis." The second of these chapters is, perhaps, the most important one in the volume. In it the author discusses with much ability, the influence of climate and atmospheric conditions as causes of Phthisis. Copious statistical tables are adduced in reference to the prevalence of the disease in various countries. The same chapter includes a brief consideration of the influence of age, sex, and digestion.

Most of our readers will remember that the facts and statistics adduced by Drs. Forrey and Drake, led to the inference that climate exercised very little influence over the development of Phthisis. Their statistics were derived mostly from the registry of mortality at the various military stations. Dr. Lawson on comparing these with a careful analysis of the United States census returns, arrives at a different conclusion, which he states as follows:

"Taking all these facts into consideration, I am fully persuaded that consumption originates far less commonly in the

Southern than in the more Northern regions, and that it gradually but perceptibly diminishes from Maine to Florida. And without claiming absolute accuracy, I believe the tables compiled from the United States census are very near the truth, and will be found sufficient guides for practical purposes. Thus, the mortality from Phthisis, in the Eastern Division, may be set down at one in three hundred and twenty-eight; in the Middle Division, one in five hundred and nineteen; in the Western Division, one in eight hundred and eighty-two; and in the Southern Division, one in twelve hundred and eighty-seven. This shows the mortality from Phthisis to be three times greater in the Northern than in the Southern."

If we may rely on these deductions, the diminution in the prevalence of consumption from North to South, is by no means "gradual," but very rapid. And yet this rule is limited in its application; for Dr. Lawson, like most other writers, admits that many localities in high Northern latitudes are remarkably exempt from the prevalence of this disease; while on the other hand, some localities even between the *tropics* afford a very high ratio of its prevalence. Again, if we look to the details of the tables contained in Dr. Lawson's work, we shall find facts strongly opposed to the idea of any *gradual* diminution from North to South. Thus Maryland is represented to have *one* death from Phthisis for every 529 of her population, while Pennsylvania has only one in 641. Michigan gives one in 602, while Wisconsin gives only one in 1050. Louisiana one in 802, while Illinois gives only one in 975. The ratio in Georgia and Florida is represented as seventy-five per cent. higher than in South Carolina. Such facts show very clearly that other causes besides the ordinary elements of climate, exert a controlling influence over the development of tubercular diseases, and greatly lessen the value of all mere geographical statistics.

The Third Division of the work contains about 100 pages, devoted to the symptoms and physical signs accompanying the several stages of phthisis, with the differential diagnosis between it and inflammatory affections of the respiratory organs.

Part Fourth, on the Therapeutics of Phthisis, embraces three chapters. The first, is devoted to the treatment of Chronic Phthisis, in all its stages, together with its incidental complications.

The second, is devoted to the treatment of Inflammatory or Acute Phthisis. And the third, contains the author's views on the special questions of change of climate—sea voyages—Gestation—Topical Medication—Prognosis and Conclusions. In the precursory or forming stage of Phthisis, the author dwells with just emphasis, on the adoption of proper hygienic regulations. The choosing of such localities as afford pure and dry air; free muscular exercise out of doors; and a nutritious diet, are conditions absolutely *essential*, to afford even a chance of recovery.

As special remedial agents, in this stage, he recommends tonics, the most important of which are the preparations of iron; cod-liver oil; and in some cases, alcoholic stimulants.

In reference to the question of the *curability* of Phthisis, the author says: "The present state of science justifies the assumption that tubercular exudation is susceptible of absorption; and furthermore, that tubercular consolidations may liquify and return to the circulation, or be eliminated through the bronchial tubes, leaving, in either case, a condition of actual cure."

Again he says: "But the cases of *permanent* cure are sufficiently numerous to become an important element in prognosis, and to assure the physician that Phthisis is, to a certain extent, a curable disease. * * * * *

"The conditions which justify a favorable prognosis may be thus stated:

"1. When the tubercles are limited to one lung, are not very extensive, and have not been associated with inflammation, either as a sequence or an inducing cause.

"2. The general health remaining in a fair condition, without rapid emaciation, fever, or derangement of digestion.

"3. A hereditary tendency to phthisis being slight or entirely absent.

"4. The patient possessing naturally a good constitution, with a sanguineous or nervo-sanguineous temperament.

"5. The occupation being favorable, or at least, not of a character to induce Phthisis, or the patient being in a condition to make a change to a more suitable business.

"6. The patient having confidence in his medical attendant, and a willingness to submit to treatment, and the ability to avail himself of all incidental means and conditions favoring his recovery, including a change of climate.

"7. A cheerful and hopeful mental constitution, and a desire to contribute his share to the successful treatment."

In looking over the work of Dr. Lawson, we find some views relating both to the Pathology and Therapeutics of Phthisis, from which we are compelled to dissent, and should time permit, we shall certainly call the attention of our readers to them at no distant day.

Neither have we found in the pages of his work, as much evidence of original investigation, as we had hoped, and yet we have perused the work with pleasure and profit. It presents a pretty full and fair summary of existing knowledge on the subject of which it treats, and it will at once take its place as a standard work, which should be in the library of every practitioner.

